# Similarities and Differences Between Eye and Mouse Dynamics During Web Pages Exploration

**DOI:** 10.3389/fpsyg.2021.554595

**Published:** 2021-03-24

**Authors:** Alexandre Milisavljevic, Fabrice Abate, Thomas Le Bras, Bernard Gosselin, Matei Mancas, Karine Doré-Mazars

**Affiliations:** ^1^Vision Action Cognition Laboratory, Psychology Institute, Université de Paris, Boulogne-Billancourt, France; ^2^Information, Signal and Artificial Intelligence Laboratory, Numediart Institute, University of Mons, Mons, Belgium; ^3^Research and Development Department, Sublime Skinz, Paris, France

**Keywords:** eye movement, behavior, computer mouse, scroll, web page

## Abstract

The study of eye movements is a common way to non-invasively understand and analyze human behavior. However, eye-tracking techniques are very hard to scale, and require expensive equipment and extensive expertise. In the context of web browsing, these issues could be overcome by studying the link between the eye and the computer mouse. Here, we propose new analysis methods, and a more advanced characterization of this link. To this end, we recorded the eye, mouse, and scroll movements of 151 participants exploring 18 dynamic web pages while performing free viewing and visual search tasks for 20 s. The data revealed significant differences of eye, mouse, and scroll parameters over time which stabilize at the end of exploration. This suggests the existence of a task-independent relationship between eye, mouse, and scroll parameters, which are characterized by two distinct patterns: one common pattern for movement parameters and a second for dwelling/fixation parameters. Within these patterns, mouse and eye movements remained consistent with each other, while the scrolling behaved the opposite way.

## 1. Introduction

Websites, and more particularly web pages, refer to a type of stimulus we potentially see every day. Such stimuli are rarely entirely visible, hence the fact that we cannot fully explore them using only our eyes. That is one of the reasons web browsing on a desktop computer requires the use and coordination of the eyes and the computer mouse. On the one hand, the eyes are used to explore and extract information of interest, such as the location of items. On the other hand, the mouse is used to interact with the content. This interaction can take multiple forms, including clicks, scrolling, and drags and drops. While clicks and drags and drops allow the user to perform actions on the visible content, scrolling drives which part of the web page is displayed. These characteristics specific to web pages induce more complex behaviors, as well as more challenging issues to address. One particularly interesting aspect is how the mouse relates to the eyes.

Eye movements have been extensively studied. For instance, we know that a fixation last in average 250–350 ms (Mackworth and Morandi, 1967; Yarbus, 1967) and that visual exploration is modulated by bottom-up and top-down factors regardless of the stimulus type (Yarbus, 1967; DeAngelus and Pelz, 2009; Helo et al., 2014; Itti and Borji, 2015). Bottom-up factors are characterized by low-level features of the stimulus, such as luminance, contrast, or edges (Tatler and Vincent, 2008), while top-down factors are characterized by high-level properties representing cognitive processes (Henderson and Hollingworth, 1999). It is generally assumed that the interaction between bottom-up and top-down factors influence how we orient our visual attention (Theeuwes and Failing, 2020). In that sense, top-down factors are usually addressed as factors influencing bottom-up ones and are not considered as totally distinct factors (Theeuwes and Failing, 2020). Furthermore, Still and Masciocchi (2010) pointed out that most of web-specific biases were top-down and were mainly related to learned behaviors. Web pages often follow a similar template: a header with main sections of a website, a content with left or right bar, and a footer at the end of the web page. Thus, users developed strategies to maximize their efficiency in visual exploration (Buscher et al., 2009). Nielsen (2010) observed that users tend to spend more time on the left part of a web page than on the right one. He also observed this behavior on right-to-left reading web pages. A more recent study from Fessenden (2017) showed a similar behavior on search engine result pages (SERP). Nielsen (2006) ran a usability experiment during which he analyzed which part of a web page users were looking at. He observed a recurring viewing pattern in the shape of the F letter. People started their browsing at the top-left corner of the web pages and read horizontally, then they were scrolling down to read a second time horizontally to finally scan the content vertically. Both factors have been widely investigated during website exploration in order to better understand user behavior and thus improve the usability of web pages. For instance, Pan et al. (2004) showed differences in visual exploration depending on the type of website, their presentation order and the gender of the user. They did not find any difference between a memorization and a free viewing task, highlighting the importance of adapting a website to its targeted audience. In his work, Tullis (2007) found that older users spent more time looking at a page content, especially navigational areas, compared to younger users. Additionally, Roth et al. (2013) showed that user expectations had an influence on visual exploration, and, more particularly, less fixations were needed to find items in expected locations compared to unexpected ones.

These studies clearly show an influence of bottom-up and top-down factors. However, Tatler and Vincent (2008) and Anderson et al. (2015) show that bottom-up influence was higher at the beginning of visual exploration. Thus, both factors alternatively influence visual exploration (Henderson, 2003; Torralba et al., 2006). As such, Cronin et al. (2020) encouraged the need to focus more on the dynamic of eye movements. They showed that the study of global eye movement parameters could not necessarily be used to distinguish different experimental conditions. To do so, they compared fixation durations and saccade amplitudes between a memorization task and an esthetic judgment task. While they did not find differences in the mean level analyses, the use of temporal and distributional analyses allowed them to discriminate the two tasks.

Previous research already highlighted the dynamic of eye movements (Unema et al., 2005; Pannasch et al., 2008; Pannasch and Velichkovsky, 2009). They found that the amplitude of saccades decreased while the duration of fixations increased over time. Pannasch and Velichkovsky (2009) and Velichkovsky et al. (2002) defined two visual exploration modes based on the relationship between saccade amplitudes and fixation durations. The ambient mode corresponds to short fixations (<180 ms) followed by saccades with an amplitude >5°, while the focal mode corresponds to long fixations (>180 ms) followed by saccades with an amplitude of <5°. Generally, visual exploration begins in ambient mode before gradually switching to focal mode (Velichkovsky et al., 2002; Pannasch and Velichkovsky, 2009). Our knowledge on these visual modes is growing but still incomplete. For instance, we know that a fixation last in average 250–350 ms (Mackworth and Morandi, 1967; Yarbus, 1967) and that visual exploration is modulated by bottom-up and top-down factors regardless of the stimulus type (Yarbus, 1967; DeAngelus and Pelz, 2009; Helo et al., 2014; Itti and Borji, 2015). A closer understanding of these two modes could help to better grasp the dynamic of eye movements when looking at complex stimuli, such as web pages. More specifically, in addition to eye movements, it would also be of interest to use these two visual modes to investigate the dynamic of mouse movements.

To our knowledge, despite the fact that the use of the computer mouse is well studied, its dynamic is rarely considered. Generally, research on the computer mouse focuses on how mouse movements could reveal users' intentions. Its availability and its potential for scalability enable innovative applications, such as authentication (Zheng et al., 2011), the prediction of the users' cognitive load (Rheem et al., 2018), the prediction of users' intentions (Guo and Agichtein, 2010; Fu et al., 2017), or pattern behavior analysis (Tzafilkou and Protogeros, 2018). One of the most studied topics is the computer mouse movement patterns commonly used by participants when browsing. Tzafilkou and Protogeros (2018) reviewed six patterns: the straight pattern (Griffiths and Chen, 2007), the hesitation pattern (Mueller and Lockerd, 2001), the horizontal reading pattern (Rodden et al., 2008), the vertical reading pattern (Rodden et al., 2008), the random pattern (Ferreira et al., 2010), and the fixed pattern (Griffiths and Chen, 2007).

Whether it is necessary to describe mouse movement patterns or their dynamic, mouse movements are not limited to moving the mouse and include scrolling as well. However, contrary to mouse movements, scrolling behavior has, to our knowledge, not been closely examined. For instance, Liu et al. (2017) investigated users' strategies when navigating SERP through their scrolling behavior. An SERP consists of a list of links corresponding to a query entered by a user in a search engine. Liu et al. (2017) analyzed the number of scrolls and their direction. In their work, Braganza et al. (2009) evaluated user preferences depending on the web page layout and the scrolling mechanism using the number of scrolls and their total duration. More generally, these studies show that the mouse is a convenient and cheap way to infer users' cognitive processes, such as intentions or reading strategies. However, these studies mostly focus on users' strategies and do not tackle quantitative analyses of mouse and scroll parameters. Such extensive statistical description could provide a baseline of typical behavior when exploring web pages and could be used to assess more precisely strategies or any other behavior.

These limitations can also be found when it comes to the relationship between the eye and the computer mouse. To this day, one of the most studied web stimuli for investigating this relationship is the SERP. On this type of web page, the coordination between the eyes and the computer mouse is higher for the vertical axis of the screen than for the horizontal axis (Rodden and Fu, 2007; Guo and Agichtein, 2010). However, this relationship remains uncertain, considering that the mouse could be used as a means to mark a potential result previously located with the eyes (Rodden et al., 2008). Furthermore, the amount of time spent by a user on an SERP can affect the location of the gaze and the mouse during the exploration (Huang et al., 2012). Navalpakkam et al. (2013) designed a model to predict the location of the eyes based on the mouse location and showed that the correlation between the eyes and the mouse is nonlinear and user dependent. More specifically, this correlation has been found for time periods during which a user looked at an area of interest (AOI) and when switched between AOIs. However, SERPs are not representative of the web and remain transitional web pages to access a content on a different website. As a matter of fact, users spend a significant cumulative amount of time on SERPs, but in short bursts of time. When focusing on common web pages, the eyes and the mouse are also coordinated on the vertical axis, and the scroll speeds influence the position of the eyes during scrolling (Milisavljevic et al., 2018). The participant is looking at the opposite part of the screen when scrolling at a high speed. Moreover, the presence of the cursor in a region of the screen correlates with the probability that the participant is fixating on this region (Chen et al., 2001). To better estimate if the eyes and the mouse are coordinated, Boi et al. (2016) generalized the work of Navalpakkam et al. (2013) by defining that the eyes and mouse must be positioned over the same content. This new definition allowed them to improve the predictive power of the models of Guo and Agichtein (2010) and Huang et al. (2012) when applied to classic web pages. Finally, when it comes to the coordination of the eyes and scrolling, web pages are not of primary interest. That is why, to our knowledge, no studies tackle the coordination between the two outside the reading field (Kumar et al., 2007; Sharmin et al., 2013).

The goal of our study was to contribute to this growing area of research by exploring the similarities and differences between movement of the eyes and computer mouse on web pages. First, we introduced a new segmentation threshold in order to differentiate two mouse movements or scrolls as precisely as possible. Then, with this new segmentation, analyses from eye movement methodology were applied to mouse movement and scrolling parameters. This methodology allowed us to investigate the influence of the tasks (free viewing and visual search) on eye, mouse, and scroll parameters. Beyond these global analyses, we also considered the influence of time on the dynamic of each type of movement through visual exploration modes.

## 2. Materials and Methods

### 2.1. Participants

We recruited 151 participants (127 females and 24 males) aged between 18 and 56 (*M* = 22.77, *SD* = ±6.33). Participants reported normal or corrected to normal vision and were naive about the purpose of the study. They were right-handed or accustomed to using a computer mouse with the right hand. A majority were undergraduate students from the psychology institute at the Université de Paris. Participants were compensated either by course credit or a 15 euro gift card. All procedures performed in studies involving human participants were in accordance with the ethical standards of the institutional and/or national research committee (local Ethics Committee of Paris Descartes University, No. CER-PD: 2018-77) and with the 1964 Helsinki declaration and its later amendments or comparable ethical standards. All subjects gave written informed consent.

### 2.2. Apparatus

Eye movements were recorded using an Eye-Link 1000 Plus (SR Research Ltd., Canada) at a 1,000 Hz sampling rate with 0.05°precision. We recorded the right eye of the participants with a 35 mm monocular lens. Mouse movements were recorded with a standard USB optical mouse with a 125 Hz polling rate. Stimuli were displayed on a 24.5 inch LCD computer screen with a 1,920 × 1,080 pixel resolution and a 144 Hz refresh rate. The experiment was run using Python 2.7 with Pylink from the manufacturer and Chromium 64.

### 2.3. Stimuli

**Figure 1:**
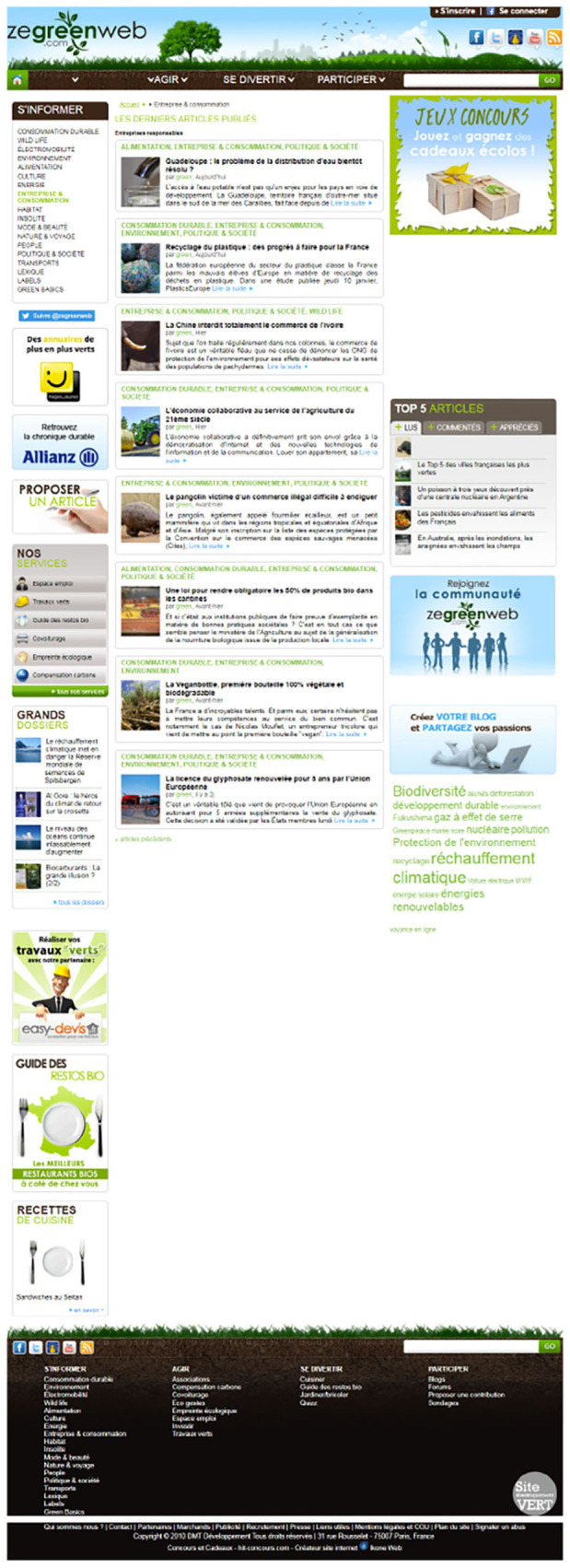
Example of website displayed during the experiment.

In this experiment, 18 web pages (see example in Figure 1) from 18 different websites were randomly presented to the participants. The web pages had a width of 1,920 pixels and their total height was between 5,000 pixels and 19,230 pixels (*M* = 6, 405*px*, *SD* = ±2, 673*px*). Participants were allowed to freely move the mouse, scroll, or click. However, hyperlinks and content animations were deactivated, thus participants could not leave the displayed web page. The presented web pages and their topic were arbitrarily chosen, including blogs, front pages, textual pages, articles (see example in Figure 1). We ensured that each selected web page followed several criteria to minimize biases. The first criteria was the language of the website. We ensured stimuli were from French websites.The second criterion was about the websites' news content. Since this study was run over several months, a web page could not have any content referring to current events or content related to a season, date, holiday, celebration, etc. As the third criterion, we checked that the web pages did not have any external advertising. In contrast to the first three criteria, which were respected on all web pages, the following criteria were counterbalanced between web pages. As Bruyer et al. (1987) explained, faces are handled differently by our brain during visual exploration. To this end, we made sure that we keep a balance of faces between the web pages. We also made sure that a balance was maintained for images, texts, general layout, and total length of the web page to have stimuli with different content types and organization. Finally, as described in the following paragraph, we gave targets already present within the original web page. Thus, we checked the number of targets available on the web page and their distribution across the page.

### 2.4. Tasks

Participants had to perform a total of nine free viewing tasks and nine visual search tasks randomly distributed on the 18 websites following a uniform distribution. Thus, each participant executed one task per web page. The balance of tasks per web page was ensured before any analyses. During the free viewing task, the participants were instructed to explore the web page freely for exactly 60 s. This duration was chosen after multiple trials and errors to provide enough data for the study of long browsing. Thus, participants had enough time to fully explore the web page. In the visual search task, participants were asked to find a target in an arbitrarily maximum of 2 min. The participants did not know how many targets there were but we informed them that there were up to three targets, with at least one, per web page. As previously defined, the targets were icons or images present on the original web page. Moreover, the targets were equally distributed between the top, middle, and bottom of the web page, and could be found on the sides, or in the content, header, or footer.

### 2.5. Procedure

**Figure 2:**
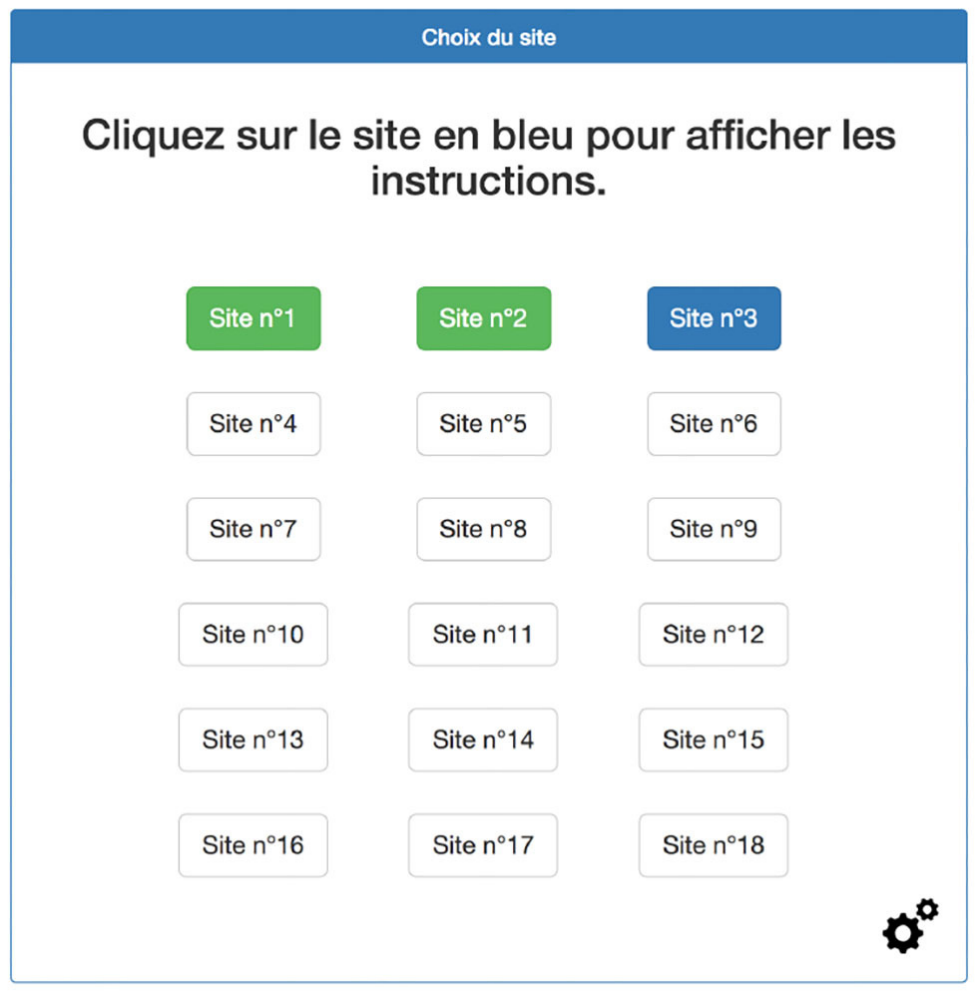
Example of screen on which participants had to click the next item to get the instruction. The white button indicates a website not yet visited, the green button a website already visited, and the blue button the next website to visit. Only the blue button was clickable.

In a quiet room, with constant luminosity, the participants were instructed to position their head on a chin rest in front of a computer screen at a viewing distance of 57 cm. The experiment then began with practice trials, one for each task. After this phase, the participants' right eye was calibrated at nine points and this was repeated until the error value was below 1°. Once the calibration was successfully complete, the participants had to click on the next trial with the mouse on a 3 × 6 table, as shown in Figure 2. Then the instructions were displayed on a new screen with a button to launch the trial. The position of this button was randomly chosen in order to avoid bias related to the first fixation commonly being at the same position as the button launching the trial. Furthermore, to ensure the web page would have completely loaded before the trial started a 3-s countdown was added to the button launching the trial. The countdown only began after the page entirely loaded, thus visual elements displayed after few seconds could be avoided. During this phase, the participants were informed of the presence of maximum three targets when carrying out the visual search task. After clicking on the button, the web page was displayed for 60 or 120 s, depending on the task. During the visual search task, the participants had to click on the targets when they founded them. If the image clicked was one of the targets, a green rectangle surrounded the target to indicate that one of the targets had been found. The participants were instructed to press the space bar on the keyboard when they thought they had found all the targets. After 1 min of the free viewing task, and 2 min or after the space bar was pressed in the visual search, the recording was stopped, and the 3 × 6 table displayed at the beginning was displayed again. Between each trial a 5-point calibration was performed. A 9-point calibration was initiated after the ninth trial, or if any problems occurred during the experiment.

### 2.6. Data Analysis

#### 2.6.1. Data Cleaning

Data from 12 participants who did not finished the experimental protocol due to calibration problems were discarded. Among the remaining 139 participants (2,502 trials), due to problems encountered during the experiment, such as calibration problems, participants talking during a trial, external noise, etc., we removed 4.88% of all trials (122 trials). The remaining data (2,380 trials) was then pre-processed and cleaned in three steps. The first step was only applied to the visual search task. The two last seconds of recording were removed in order to deal with the moment the participant looked at the keyboard when pressing the space bar. In addition, and for the same reason, residual fixations below the screen at the end of the exploration were removed. Throughout the second step, blinks and fixations under 100 ms around a blink were cleaned (Holmqvist et al., 2011). During the third and final step, fixations with a visual angle of more than 3° from the screen's border were deleted. Fixations outside the screen, but below the 3° threshold, were reset to the corresponding border of the screen. These three steps led to deletions within all the trials. All 139 participants, and 95% of the initial trials (2,378 trials), were kept. In total, 91.74% of all records were retained for analyses. Finally, only the first 20 s were selected for this work, and 18 more trials were deleted due to insufficient mouse moves or scrolling events (2,360 trials remaining). It should be noted that eye movement analyses were run on aggregated data, and scrolling and mouse events on raw data. All analyses were carried out using Python 3.6.

#### 2.6.2. Events Segmentation

There are a number of well-established, and ever improving, methods to label raw data from eye recordings. However, mouse and scroll recordings lack such a method, specifically to differentiate two close events. While it is easy to determine if two mouse or scroll events separated by 2 or 3 s are indeed two distinct events, doing the same operation for 2 events with, for instance, <1 s in between is much difficult.

In the literature, we can find multiple attempts to define a threshold allowing the differentiation of idle time and movement of the mouse. Since the mouse is a pointing device, a simple threshold seems to be appropriate, contrary to eye movements that are more complex. In their attempt to define a new behavioral biometric technique based on mouse movements, Gamboa and Fred (2004) differentiated two mouse movements as a pause in the user's interaction when the two consecutive events were separated by more than 100 ms. In their work, Reeder and Maxion (2006) arbitrarily considered a threshold of 3 s with to the user being silent and inactive (with both the mouse and the keyboard) in order to propose a method to detect user difficulties when using an interface. On the other hand, Feher et al. (2012) empirically set this threshold to 500 ms to categorize mouse movements and thus uniquely identify users. More recently, Seelye et al. (2015) studied cognitive impairment using computer mouse movement patterns. They mentioned a median idle time, which is the time spent idling or pausing between mouse movements, of 310 ms. In the continuity of the work of Gamboa and Fred (2004), Antal and Egyed-Zsigmond (2019) used a threshold of 10 s to segment mouse movements and used them to detect intruders on a computer.

Moreover, several studies focused specifically on scroll segmentation (Braganza et al., 2009; Brady et al., 2018; Milisavljevic et al., 2018). In their study into the scrolling behavior, Braganza et al. (2009) determined that two scrolls recorded within 1 s of each other were considered as a single scroll. To set this threshold, they tried values ranging from 200 ms to 4 s, with increments of 100 ms. They did not find any major differences between these timings, and consequently chose 1 s as a threshold. In their study, Milisavljevic et al. (2018) defined a scroll session as a set of continuous scroll events ended with a mouse movement. On the topic of scrolling when reading, Brady et al. (2018) sampled a frame every 100 ms to check if the displayed text had moved. If it had moved more than half a line between one sentence and the next, it was counted as a scroll. Even though the presented techniques try to segment scrolling or mouse events, these techniques are mostly based on arbitrary thresholds. Thus, our goal is to propose a better approach of mouse and scroll events segmentation to provide more robust analyses.

If we take a closer look at our previous attempt to segment events, we defined a threshold based on the events number rather than the time (Milisavljevic et al., 2018). This definition does not take into account all parameters that come into play when interacting using mouse or scroll. The main parameter is the fact that, on a desktop, it is possible to move the mouse during a scroll. In such a case, a single scroll would be labeled as two different scrolls. The bias will remain if the participant uses the browser scroll bar, which allows the user to grab a bar on the right of the browser and scroll by moving it up or down. Furthermore, Brady et al. (2018) used a spatial threshold of 40 pixels to identify when a user was scrolling, but this is applicable to mouse movements. In addition to highlighting the need to use a time-based threshold, all previously mentioned studies did not correctly handle stops and micro-stops. A stop is a period of time during which the user does not move the mouse or scroll. During this idle time, the user explores the web page and processes it. But based on this definition, a new question arises: what is the minimal length of this period of time to give the user enough time to process the stimulus and make the decision to keep moving, scrolling, or stop entirely? In other terms, how can we differentiate micro-stops from the movement itself? A micro-stop is an interruption during the action which is long enough to allow the user to make a decision, but this is not visible to the eye. To differentiate micro-stops from movements, we looked at the study from Moher and Song (2019) in which they compared behaviors between a 3D reach tracker, a computer mouse, and a stylus. Among multiple conditions, they measured the average response latency of 220 ms when displacing a target. This could be considered as the minimum time to visualize a target's new position and make the decision to reorient the movement. Thus, a micro-stop could not be <220 ms, and a stop below this threshold should be considered as the continuity of the previous action. We used a unified threshold to segment mouse movements and scrolls. We chose a threshold of 300 ms to differentiate two distinct movements or scrolls. This corresponds to the average visual fixation duration in a scene viewing (Henderson and Hollingworth, 1998). Despite the fact that visual fixations can be shorter than 300 ms, this does not apply to ecological conditions and semantic-rich stimuli, such as web pages.

#### 2.6.3. Variables

After all cleaning processes, we ran our analyses on a wide range of new parameters. In the state-of-the-art, the same types of parameters are frequently used. For the use of the mouse, these include curvature, trajectory, clicks, dwells, or the number of movements (Zheng et al., 2011; Fu et al., 2017; Rheem et al., 2018; Tzafilkou and Protogeros, 2018), and for scrolls, amplitude, speed, and number (Braganza et al., 2009; Liu et al., 2017; Milisavljevic et al., 2018). In comparison, eye-mouse studied parameters are more related to their respective positions, but are not limited to this factor. For instance, eye-mouse distance, content hovered, lag, percentage of regions visited by both the eyes and mouse, etc., have been used to study their relationship (Chen et al., 2001; Rodden and Fu, 2007; Rodden et al., 2008; Guo and Agichtein, 2010; Huang et al., 2012; Navalpakkam et al., 2013; Boi et al., 2016).

In this paper, we propose a more complete set of parameters directly inspired from eye movement analyses. These parameters include dwell duration, movement duration, movement amplitude, and number of events. It should be noted that duration variables are expressed in seconds or milliseconds, while amplitude variables are expressed in degrees of visual angle. Furthermore, in order to better characterize the dynamic of the exploration through ambient and focal visual modes, we apply, for the first time, the K coefficient defined by Krejtz et al. (2016) to mouse and scroll events. This coefficient is calculated by averaging the differences in z-scores between the duration of each fixation and the next saccade, as shown in Equation (1). A negative value indicates that the fixation *d*_*i*_ is short and the next saccade *a*_*i*+1_ is long (>5°). In contrast, a positive value suggests that the fixation *d*_*i*_ is long and the next saccade *a*_*i*+1_ is short (<5°) which corresponds to a focal mode.

(1) $$\begin{array}{l} K=\frac{1}{n}\underset{n}{\sum }\frac{d_{i}-\mu_{d}}{\sigma_{d}}-\frac{a_{i+1}-\mu_{a}}{\sigma_{a}} \end{array}$$

Milisavljevic et al. (2019) introduced two new variables to better capture the dynamic of focal and ambient modes. While the K coefficient did not discriminate between the different stimuli used in their study, the number of switches between modes did. It is for this reason that we are using these parameters to more precisely describe the dynamic of the exploration for both the eyes and mouse.

#### 2.6.4. Mouse and Scroll Overlap

Participants were able to independently move the mouse and scroll. Consequently, this led to overlaps between mouse movements and scrolls. We found that this overlap occurred only 10% (*SD* = ±4.83%) of the total mouse movement time and 15% (*SD* = ±10.59%) of the total scrolling time. During these overlaps, we observed mouse movements with an amplitude of 0.02°(*SD* = ±0.02°) and a duration of 240 (*SD* = ±195.53*ms*) for a total duration of 570 (*SD* = ±430*ms*). As described, during overlaps, movements represented a negligible part of the exploration. Moreover, these overlaps followed three main patterns: move–scroll, scroll–move, and move–scroll–move. The move–scroll pattern refers to a scroll that began while already moving the mouse. This pattern occurred 43% of the time and was the most frequent. The second pattern we observed was the scroll–move pattern. This pattern is the exact opposite: the participant began to move the mouse while already scrolling. This pattern happened 25% of time. The move–scroll–move pattern is when the participant scrolled within a single mouse move. This was less common and occurred 21% of the time. Finally, the 11% remaining was exotic patterns, such as move–scroll–move–scroll or move–scroll–move–scroll–move, which represent 2% each. Due to the low frequency of overlaps between scrolls and mouse movements, we can safely conclude that these specific movements are residual movements or involuntary micro-movements generated by the use of the mouse wheel. For this reason, we did not take overlaps into account in the following analyses.

## 3. Results

To study the similarities and differences between eye movements, mouse movements, and scrolling, we ran two types of analyses. We first described eye, mouse, and scroll parameters globally, to clearly define what a mouse or scroll movement was, and summarized them in Table 1. Then, we examined the role of tasks and time, by performing a 2 (free viewing and visual search) X 4 (0–5 s time-bin, 5–10 s time-bin, 10–15 s time-bin, and 15–20 s time-bin) repeated measures analyses of variance (ANOVAs). *Post-hoc* analyses were run using the pairwise Student's *t*-test with a Bonferroni correction. It should be noted that only mouse and scroll movement parameters are presented in this section (see Table 1 for dwell parameters). Contrary to a fixation that provides information of current cognitive processes, a dwell generally means that the mouse have not been used. Moreover, the duration of a dwell is much longer than a fixation and can easily last the equivalent of 10 fixations. This difference of scale does not make it possible to determine what falls within the scope of the cognitive process in progress, or the simple nonuse of the mouse. However, movement parameters remain comparable.

**Table 1:** Global means and standard deviations of all studied variables (139 participants on 18 web pages for 20 s each).

	**Eye**		**Mouse**		**Scroll**	
	**Mean**	**Std**.	**Mean**	**Std**.	**Mean**	**Std**.
**Fixations/Dwells**						
% of time	85.76%	±1.72%	79.15%	±8.33%	83.42%	±5.32%
Avg. count	72.18	±6.5	8.71	±2.57	10.07	±1
Avg. duration	236.97 ms	±24.45 ms	2.49 s	±1 s	2.3 s	±0.71 s
Tot. duration	16.83 s	±0.33 s	15.68 s	±1.65 s	16.52 s	±2.33 s
**Movements**						
% of time	14.24%	±1.72%	20.85%	±8.33%	16.58%	±5.32%
Avg. count	72.18	±6.5	6.04	±1.78	8.77	±2.04
Avg. duration	39.04 ms	±4.29 ms	768.24 ms	±342.55 ms	367.57 ms	±121.65 ms
Tot. duration	2.8 s	±0.34 s	4.13 s	±1.65 s	3.28 s	±1.39 s
Avg. amplitude	6.10°	±0.67°	0.27°	±0.23°	8.52°	±2.35°
Tot. amplitude	435.22°	±52.74°	1.6°	±0.71°	70.79°	±19.70°
**Dynamic**						
Avg. K coeff.	−0.13	±0.2	−0.35	±0.63	0.43	±0.45
Avg. nb. switches	33.15	±3.25	3.78	±0.89	3.63	±0.74
% time in ambient	42.82%	±6.81%	–	–	–	–
% time in focal	57.37%	±6.8%	–	–	–	–

### 3.1. Eye Movements Analysis

We measured a stable distribution of fixations and saccades across the different conditions. During the exploration of a website, participants spent approximately 14% (*SD* = ±1.72%) of the time making a saccade (see Table 1). Although this proportion was maintained across the tasks, we found a task effect on the distribution of fixations/saccades [*F*_(1,138)_= 231.98, *p* < 0.001]. Participants spent 13.6% (*SD* = ±1.79%) of the time making a saccade in the free viewing task and 15% (*SD* = ±1.84%) during the visual search task. Furthermore, we found a time effect [*F*_(3,414)_= 685.59, *p* < 0.001] present between the first and second time-bins (*t* = −29.50, *p* < 0.001), and between the second and third time-bins (*t* = 8.98, *p* < 0.001), but not between the third and fourth time-bins (*t* = −2.33, *p* > 0.05). We also found a significant interaction effect between task and time [*F*_(1,138)_= 3.48, *p* < 0.05], and *post-hoc* analyses confirmed that main effects were preserved (see Table 2).

**Table 2:** Means and standard deviations of all studied variables as a function of tasks and time-bins for the eye (139 participants on 18 web pages for 20 s each).

	**Free viewing**				**Visual search**				
	***T*1_*F*_**	***T*2_*F*_**	***T*3_*F*_**	***T*4_*F*_**	***T*1_*V*_**	***T*2_*V*_**	***T*3_*V*_**	***T*4_*V*_**	***Non-significant***
**Fixations/Dwells**									
Amount of time (%)	84.06, 2.10	86.69, 1.92	87.20, 1.93	87.47, 1.83	82.90, 2.11	85.13, 2.07	85.90, 1.92	85.91, 1.95	[*T*3_*F*_-*T*4_*F*_, *T*3_*V*_-*T*4_*V*_]
Avg. count	18.93, 1.83	18.93, 1.87	18.71, 1.94	18.60, 1.87	17.39, 1.62	17.32, 1.74	16.98, 1.79	16.70, 1.74	[*T*1_*F*_-*T*2_*F*_, *T*2_*F*_-*T*3_*F*_, *T*3_*F*_-*T*4_*F*_, *T*1_*V*_-*T*2_*V*_, *T*3_*V*_-*T*4_*V*_]
Avg. duration (s)	211.27, 23.27	234.24, 28.06	238.83, 30.34	241.28, 30.33	227.61, 23.75	251.11, 30.66	261.18, 33.71	262.53, 33.60	[*T*3_*F*_-*T*4_*F*_, *T*3_*V*_-*T*4_*V*_]
**Movements**									
Amount of time (%)	15.94, 2.10	13.31, 1.92	12.80, 1.93	12.53, 1.83	17.10, 2.11	14.87, 2.07	14.10, 1.92	14.09, 1.95	[*T*3_*F*_-*T*4_*F*_, *T*3_*V*_-*T*4_*V*_]
Avg. count	18.93, 1.83	18.93, 1.87	18.71, 1.94	18.60, 1.87	17.39, 1.62	17.32, 1.74	16.98, 1.79	16.70, 1.74	[*T*1_*F*_-*T*2_*F*_, *T*2_*F*_-*T*3_*F*_, *T*3_*F*_-*T*4_*F*_, *T*1_*V*_-*T*2_*V*_, *T*3_*V*_-*T*4_*V*_]
Avg. duration (ms)	39.51, 5.14	35.28, 4.81	34.39, 4.85	33.91, 4.74	46.07, 4.99	42.86, 4.97	41.58, 4.71	41.83, 5.21	[*T*3_*F*_-*T*4_*F*_, *T*3_*V*_-*T*4_*V*_]
Avg. amplitude (°)	6.26, 1.03	4.97, 0.88	4.61, 0.89	4.47, 0.88	8.41, 0.97	7.32, 0.96	6.82, 0.96	6.84, 1.01	All
**Dynamic**									
Avg. K coeff.	−0.36, 0.25	0.05, 0.25	0.15, 0.26	0.20, 0.26	−0.62, 0.25	−0.24, 0.28	−0.08, 0.31	−0.07, 0.29	All
Avg. switch nb.	8.85, 1.11	9.31, 1.36	8.90, 1.48	8.89, 1.43	7.79, 1	8.70, 1.18	8.53, 1.18	8.28, 1.24	[*T*3_*F*_-*T*3_*V*_, *T*3_*F*_-*T*4_*F*_, *T*2_*V*_-*T*3_*V*_, *T*3_*V*_-*T*4_*V*_]
% time in ambient (%)	52.27, 8.96	37.01, 8.38	33.36, 9.14	32.02, 8.75	59.82, 8.30	47.93, 9.12	43.75, 9.46	44.03, 8.92	[*T*3_*F*_-*T*4_*F*_, *T*3_*V*_-*T*4_*V*_]
% time in focal (%)	48.51, 8.91	63.67, 8.34	67.32, 9.09	68.64, 8.72	41.14, 8.26	52.92, 9.10	57.10, 9.43	56.83, 8.88	[*T*3_*F*_-*T*4_*F*_, *T*3_*V*_-*T*4_*V*_]

*The non-significant column regroups all post-hoc with a p-value > 0.05. Not mentioned post-hocs have a p-value*.

#### 3.1.1. Number of Fixations and Saccades

Globally, participants made an average of 72 (*SD* = ±6.5) fixations and saccades during the exploration of a website for 20 s. The task had an effect on the number of fixations and saccades [*F*_(1,138)_ = 424.29, *p* < 0.001] with less fixations and saccades during the visual search (*M* = 68.4, *SD* = ±6.31) compared to the free viewing task (*M* = 75.16, *SD* = ±7.08). We found a time effect [*F*_(3,414)_ = 27.86, *p* < 0.001], but there were no significant differences between the first and second time-bins (*t* = 0.32, *p* > 0.05). However, there was a significant decrease in the number of fixations and saccades between the second and third time-bins (*t* = −4.84, *p* < 0.001), as well as between the third and fourth time-bins (*t* = −2.85, *p* < 0.05). The interaction between the time and task was also significant [*F*_(3,414)_ = 3.29, *p* < 0.05]. The main task effect was maintained for each time-bin (all *p <0.001*). In free viewing task, there were no significant differences between the successive time-bins (all *p >0.05*). However, in visual search, the only difference with the main time effect was the absence of a reduction between the third and fourth time-bins (*p >0.05*) (see Table 2).

#### 3.1.2. Fixation Duration

We observed an average fixation duration of 236 ms (*SD* = ±24.45*ms*). The average fixation duration varied according to the task [*F*_(1,138)_ = 195.75, *p* < 0.001]. Fixations were shorter during the free viewing task (*M* = 229*ms*, *SD* = ±24.59*ms*) than during the visual search task (*M* = 247.17*ms*, *SD* = ±26.41*ms*). The average fixation duration significantly increased over time [*F*_(3,414)_ = 297.65, *p* < 0.001] up to the third time-bin. More precisely, the first time-bin was significantly different from the second time-bin (*t* = 20.91, *p* < 0.001), and this second time-bin was significantly different from the third time-bin (*t* = 6.80, *p* < 0.001). However, the third time-bin was not significantly different from the fourth (*p > 0.05*. There was also an interaction effect between task and time [*F*_(3,414)_ = 3.29, *p* < 0.05], but *post-hoc* analyses confirmed that main effects were preserved (see Table 2).

#### 3.1.3. Saccade Amplitude

We measured an average saccade amplitude of 6.1 °(*SD* = ±0.67°). We found a significant difference between the tasks [*F*_(1,138)_ = 1314.42, *p* < 0.001], saccade amplitudes were shorter during the free viewing task (*M* = 5.08°, *SD* = ±0.77°) than during the visual search task (*M* = 7.36°, *SD* = ±0.77°). We also observed a time effect [*F*_(3,414)_ = 378.60, *p* < 0.001] up to the third time-bin. The average saccade amplitude decreased from the first to the second time-bin (*t* = −21.27, *p* < 0.001), and from the second to the third time-bin (*t* = −8.45, *p* < 0.001), but not between the third and fourth time-bins (*t* = −1.55, *p* > 0.05). However, there was no significant interaction between the time and task [*F*_(3,414)_ = 2.11, *p* > 0.05] (see Table 2).

#### 3.1.4. Dominant Mode

Finally, to understand the dynamic of visual exploration, we computed the K coefficient and its associated variables, as defined by Krejtz et al. (2016) and Milisavljevic et al. (2019), and described in the Materials and Methods section. Globally, we found a dominance of the ambient mode with a K coefficient below zero (*M* = −0.13, *SD* = ±0.2). There was a significant difference between tasks [*F*_(1,138)_ = 313.8, *p* < 0.001], which indicated a higher dominance of the ambient mode in the visual search task (*M* = −0.28, *SD* = ±0.23) than in the free viewing task (*M* = −0.01., *SD* = ±0.21.) We also found a significant time effect [*F*_(3,414)_ = 579.66, *p* < 0.001]. The K coefficient, beginning with negative values, got significantly closer to 0 between the first and second time-bins (*t* = −27.10, *p* < 0.001), became positive between the second and third time-bins (*t* = −10.23, *p* < 0.001), but did not significantly change between the third and fourth time-bins (*t* = 1.94, *p* > 0.05). *Post-hoc* analyses did not show a significant interaction between the task and time [*F*_(3,414)_ = 1.97, *p* > 0.05] (see Table 2).

#### 3.1.5. Visual Modes Switches

As described in the Methodology section, the number of visual modes switches corresponds to how many times a participant switched from ambient to focal and focal to ambient during a trial. Participants switched between visual modes 33.15 (*SD* = ±3.25) times and this amount varied according to the task [*F*_(1,138)_ = 63.06, *p* < 0.001]. There were more switches in the free viewing task (*M* = 34.26, *SD* = ±4.30) than in the visual search task (*M* = 31.67, *SD* = ±3.22). There was also a time effect [*F*_(3,414)_ = 22.69, *p* < 0.001]. The number of visual mode switches significantly increased between the first and second time-bins (*t* = 8.05, *p* < 0.001), but significantly decreased between the second and third time-bins (*t* = −4.05, *p* < 0.001). It was not, however, significantly different between the third and fourth time-bins (*t* = −1.24, *p* > 0.05). Furthermore, we found a significant interaction between the task and time [*F*_(3,414)_ = 6.33, *p* < 0.001]. The main task effect was maintained except for the third time-bin (*t* = 4.33, *p* > 0.05). Similarly, the main time effect was preserved for the free viewing task, but not in the visual search task, during which there were no significant differences between the second and third, and the third and fourth time-bins (all *p > 0.05*) (see Table 2).

#### 3.1.6. Visual Modes Proportions

The participants spent, in total, 43% (*SD* = ±6.81%) of the time in ambient mode. This proportion significantly varied according to the task [*F*_(1,138)_ = 358.75, *p* < 0.001]. It was higher in the visual search task (*M* = 48.35%, *SD* = ±7.33%) than in the free viewing task (*M* = 38.21%, *SD* = ±7.65%). There was a significant time effect [*F*_(3,414)_ = 638.94, *p* < 0.001]. The proportion of time spent in ambient mode significantly decreased between all successive time-bins: between the first and second time-bins (*t* = −31.30, *p* < 0.001), between the second and third time-bins (*t* = −9.32, *p* < 0.001), and between the third and fourth time-bins (*t* = −1.44, *p* > 0.05). We also found a significant interaction between the time and task [*F*_(3,414)_ = 8.75, *p* < 0.001], but *post-hoc* analyses confirmed that main effects were preserved (see Table 2).

To summarize, we found a task and time effect on all the variables of eye movements parameters. Most of the parameters increased over time to then stabilize starting at the third time-bin (after 10–15 s). More specifically, fixation-related variables increased and movement-related variables decreased over time. Moreover, ambient mode was predominant during the exploration but progressively switched to focal mode as time went by.

### 3.2. Mouse Analysis

The participants spent 20.85% (*SD* = ±8.33%) of the time moving the mouse during their exploration. We found a significant task effect [*F*_(1,138)_ = 37.66, *p* < 0.001], the proportion of time spent moving the mouse was significantly higher in the visual search task (*M* = 23.33%, *SD* = ±8.48%) than in the free viewing task (*M* = 18.94%, *SD* = ±10.11%). We also observed a time effect [*F*_(3,414)_ = 420.24, *p* < 0.001] with a significant decrease between the first and second time-bins (*t* = −24.14, *p* < 0.001), and between the second and third time-bins (*t* = −3.25, *p* < 0.01). However, there was no significant difference between the third and fourth time-bins (*t* = −1.68, *p* > 0.05). There was a significant interaction between time and task [*F*_(3,414)_ = 7.75, *p* < 0.001]. The main task effect was maintained excepted for the second time-bin (*t* = 1.2, *p* > 0.05). The main time effect was preserved in the free viewing, but not entirely during the visual search task, there was no significant difference between the second and third time-bins (*p > 0.05*) (see Table 3).

**Table 3:** Means and standard deviations of all studied variables as a function of tasks and time-bins for the mouse (139 participants on 18 web pages for 20 s each).

	**Free Viewing**				**Visual Search**				
	***T*1_*F*_**	***T*2_*F*_**	***T*3_*F*_**	***T*4_*F*_**	***T*1_*V*_**	***T*2_*V*_**	***T*3_*V*_**	***T*4_*V*_**	***Non-significant***
**Fixations/Dwells**									
% of time (%)	63.68, 12.82	79.74, 13.30	82.44, 12.71	83.85, 12.24	60.14, 12.15	79.39, 11.25	77.84, 10.76	77.70, 10.53	[*T*2_*F*_-*T*2_*V*_, *T*3_*F*_-*T*4_*F*_, *T*2_*V*_-*T*3_*V*_, *T*3_*V*_-*T*4_*V*_]
Avg. count	3.52, 0.51	3.38, 0.73	3.22, 0.66	3.13, 0.66	3.71, 0.59	3.51, 0.79	3.54, 0.70	3.57, 0.78	[*T*3_*F*_-*T*4_*F*_, *T*2_*V*_-*T*3_*V*_, *T*3_*V*_-*T*4_*V*_]
Avg. duration (s)	1.00, 0.29	1.35, 0.43	1.45, 0.43	1.52, 0.43	0.89, 0.27	1.30, 0.39	1.25, 0.35	1.24, 0.35	[*T*3_*F*_-*T*4_*F*_, *T*2_*V*_-*T*3_*V*_, *T*3_*V*_-*T*4_*V*_]
**Movements**									
% of time (%)	36.43, 12.79	22.32, 13.38	20.22, 12.96	18.31, 12.88	39.90, 12.19	22.97, 11.24	23.88, 11.01	23.96, 10.22	[*T*2_*F*_-*T*2_*V*_, *T*3_*F*_-*T*4_*F*_, *T*2_*V*_-*T*3_*V*_, *T*3_*V*_-*T*4_*V*_]
Avg. count	2.19, 0.44	2.04, 0.62	1.95, 0.62	1.79, 0.55	2.34, 0.48	2.12, 0.71	2.11, 0.59	2.15, 0.60	[*T*2_*F*_-*T*2_*V*_, *T*3_*F*_-*T*4_*F*_, *T*2_*V*_-*T*3_*V*_, *T*3_*V*_-*T*4_*V*_]
Avg. duration (ms)	965, 399	570, 431	511, 358	517, 423	1, 000, 452	572, 334	589, 328	592, 303	[*T*1_*F*_-*T*1_*V*_, *T*2_*F*_-*T*2_*V*_, *T*3_*F*_-*T*4_*F*_, *T*2_*V*_-*T*3_*V*_, *T*3_*V*_-*T*4_*V*_]
Avg. amplitude (°)	0.43, 0.27	0.15, 0.16	0.11, 0.12	0.13, 0.17	0.46, 0.31	0.18, 0.24	0.19, 0.16	0.20, 0.25	All
**Dynamic**									
Avg. K coeff.	−1.02, 0.74	−0.05, 0.71	0.34, 0.64	0.63, 0.79	−1.13, 0.81	−0.14, 0.99	0.08, 0.55	0.12, 0.64	All
Avg. switch nb.	1.61, 0.32	1.51, 0.37	1.45, 0.34	1.43, 0.37	1.65, 0.32	1.60, 0.40	1.60, 0.39	1.64, 0.38	[*T*1_*F*_-*T*1_*V*_, *T*3_*F*_-*T*4_*F*_,*T*2_*V*_-*T*3_*V*_, *T*3_*V*_-*T*4_*V*_]

*The non-significant column regroups all post-hocs with a p-value > 0.05. Not mentioned post-hocs have a p-value <0.05 or less*.

#### 3.2.1. Number of Mouse Movements

The participants did 6.04 (*SD* = ±1.78) movements on average. We found a task effect [*F*_(1,138)_ = 73.45, *p* < 0.001] with more mouse movements during the visual search task (*M* = 6.77, *SD* = ±2.01) than during the free viewing task (*M* = 5.43, *SD* = ±1.97). We found an influence of time [*F*_(3,414)_ = 183.46, *p* < 0.001] with a significant decrease between the first and second time-bins (*t* = −14.34, *p* < 0.001), and between the second and third time-bins (*t* = −4.70, *p* < 0.001). However, there was no significant difference between the third and fourth time-bins (*t* = −1.79, *p* > 0.05). We also found a significant interaction between time and task [*F*_(3,414)_ = 14.15, *p* < 0.001]. The main task effect was preserved excepted for the second time-bin (*p > 0.05*). In the free viewing task, the main time effect was preserved, but in the visual search task this main effect was maintained only between the first and second time-bins (*p <0.001*) (see Table 3).

#### 3.2.2. Duration of Mouse Movements

The participants moved the mouse for 768 ms (*SD* = ±342.55*ms*) on average. We found a task effect [*F*_(1,138)_ = 15.63, *p* < 0.001] with significantly longer mouse movements in the free viewing task (*M* = 772.68*ms*, *SD* = ±362.58*ms*) than in the visual search task (*M* = 767.43*ms*, *SD* = ±386.39*ms*). Moreover, we found a time effect [*F*_(3,414)_ = 269.83, *p* < 0.001] with a significant decrease between the first and second time-bins (*t* = −19.53, *p* < 0.001), but no significant difference between the second and third time-bins (*t* = −2.56, *p* > 0.05) or between the third and fourth time-bins (*t* = 0.74, *p* > 0.05). We also found a significant interaction between time and task [*F*_(3,414)_ = 3.69, *p* < 0.05]. However, the main task effect was preserved only for the two last time-bins (all *p <0.005*), while the main time effect was only preserved for the visual search task. During the free viewing task, we observed significant differences between the first and second time-bins, and between the second and third time-bins (all *p > 0.05*) (see Table 3).

#### 3.2.3. Amplitude of Mouse Movements

The participants performed mouse movements of 0.27°(*SD* = ±0.23°) on average. We found a significant differences between the two tasks [*F*_(1,138)_ = 24.16, *p* < 0.001]. The average amplitude slightly decreased from the free viewing task (*M* = 0.26°, *SD* = ±0.2°) to the visual search task (*M* = 0.3°, *SD* = ±0.3°). We also found a time effect [*F*_(3,414)_ = 235.57, *p* < 0.001]. There was a significant decrease between the first and second time-bins (*t* = −17.57, *p* < 0.001), but no significant differences between the second and third time-bins (*t* = −2.42, *p* > 0.05) or between the third and fourth time-bins (*t* = 0.22, *p* > 0.05). We did not find any interaction effect [*F*_(3,414)_ = 1.61, *p* > 0.05] (see Table 3).

#### 3.2.4. Dynamic of Mouse Movements

Here, K coefficient is used to better understand the mouse movement dynamic. The K coefficient showed a dominance of the ambient mode (*M* = −0.35, *SD* = ±0.63). We found significant differences between tasks [*F*_(1,138)_ = 15.27, *p* < 0.001], which was slightly higher in the free viewing task (*M* = −0.31, *SD* = ±0.58) than in the visual search task (*M* = −0.39, *SD* = ±0.77). There also was a significant time effect [*F*_(3,414)_ = 410.86, *p* < 0.001]. We found a significant increase between all successive time-bins (all *p <0.001*). However, there was no significant interaction effect [*F*_(3,414)_ = 2.48, *p* > 0.05] (see Table 3).

#### 3.2.5. Mode Switches

On average, 3.78 (*SD* = ±0.89) switches occurred between modes given by the K coefficient. There was a significant task effect [*F*_(1,138)_ = 70.08, *p* < 0.001], which was characterized by a lower number of mode switches during the free viewing task (*M* = 3.44, *SD* = ±1.04) than during the visual search task (*M* = 4.19, *SD* = ±1.07). There was also a significant time effect [*F*_(3,414)_ = 109.86, *p* < 0.001]. The number of switches significantly increased between the first and second time-bins (*t* = 11.68, *p* < 0.001), and between the second and third time-bins (*t* = 3.72, *p* < 0.005), but there was no significant difference between the third and fourth time-bins (*t* = 1.42, *p* > 0.05). We also found a significant interaction between the time and task [*F*_(3,414)_ = 11.93, *p* < 0.001]. The main task effect was preserved except for the first time-bin (*p <0.05*). Furthermore, the main time effect was maintained for the free viewing task, but, for the visual search task, the first and second time-bins were significantly different (*p <0.001*), while remaining time-bins did not have significant differences (all *p > 0.05*) (see Table 3).

To summarize, we found a task and time effect for all the mouse parameters. As found for eye movements, most of the mouse parameters stabilized at the end of the exploration. Interestingly, the mouse parameters behaved similarly to eye movements parameters. Finally, ambient mode was the prevailing mode for mouse movements, but, as for the eyes, progressively switched to the focal mode over time.

### 3.3. Scroll Analysis

The participants, globally, spent 16.58% (*SD* = ±5.32%) of a trial scrolling. There was a task effect [*F*_(1,138)_ = 469.10, *p* < 0.001]. The proportion of time spent scrolling was higher in the visual search task (*M* = 23.80%, *SD* = ±8.28%) compared to the free viewing task (*M* = 10.86%, *SD* = ±4.87%). We also found a time effect [*F*_(3,414)_ = 239.92, *p* < 0.001]. There was a significant increase between the first and second time-bins (*t* = 20.74, *p* < 0.001), as well as between the third and fourth time-bins (*t* = 3.70, *p* < 0.005), while there was no significant differences between the second and third time-bins (*t* = 0.06, *p* > 0.05). We found a significant interaction between the time and task [*F*_(3,414)_ = 11.94, *p* < 0.001]. The main task effect was maintained for all time-bins (all *p <0.001*). However, the time effect was not preserved. In both tasks, the first and the second time-bins were significantly different (*t* = −20.5, *p* < 0.001), but we did not find significant differences between other time-bins (*p > 0.05*) (see Table 4).

**Table 4:** Means and standard deviations of all studied variables as a function of tasks and time-bins for scrolling (139 participants on 18 web pages for 20 s each).

	**Free viewing**				**Visual search**				
	***T*1_*F*_**	***T*2_*F*_**	***T*3_*F*_**	***T*4_*F*_**	***T*1_*V*_**	***T*2_*V*_**	***T*3_*V*_**	***T*4_*V*_**	***Non-significant***
**Fixations/Dwells**									
Amount of time (%)	91.40, 3.51	86.19, 6.16	85.58, 5.96	84.58, 6.46	82.14, 8.23	73.54, 9.35	74.09, 8.66	72.56, 9.20	[*T*2_*F*_-*T*3_*F*_,*T*3_*F*_-*T*4_*F*_, *T*2_*V*_-*T*3_*V*_,*T*3_*V*_-*T*4_*V*_]
Avg. count	2.50, 0.45	3.24, 0.62	3.28, 0.66	3.35, 0.67	3.35, 0.73	4.36, 0.80	4.35, 0.72	4.29, 0.71	[*T*2_*F*_-*T*3_*F*_,*T*3_*F*_-*T*4_*F*_, *T*2_*V*_-*T*3_*V*_,*T*3_*V*_-*T*4_*V*_]
Avg. duration (s)	1.91, 0.29	1.50, 0.33	1.48, 0.31	1.44, 0.32	1.39, 0.39	0.95, 0.30	0.95, 0.26	0.93, 0.27	[*T*2_*F*_-*T*3_*F*_,*T*3_*F*_-*T*4_*F*_, *T*2_*V*_-*T*3_*V*_,*T*3_*V*_-*T*4_*V*_]
**Movements**									
Amount of time (%)	8.68, 3.53	13.84, 6.18	14.46, 5.94	15.45, 6.43	17.90, 8.19	26.46, 9.35	25.91, 8.66	27.58, 9.57	[*T*2_*F*_-*T*3_*F*_,*T*3_*F*_-*T*4_*F*_, *T*2_*V*_-*T*3_*V*_,*T*3_*V*_-*T*4_*V*_]
Avg. count	1.50, 0.44	2.22, 0.62	2.26, 0.64	2.33, 0.67	2.35, 0.73	3.35, 0.81	3.32, 0.72	3.26, 0.68	[*T*2_*F*_-*T*3_*F*_,*T*3_*F*_-*T*4_*F*_, *T*2_*V*_-*T*3_*V*_,*T*3_*V*_-*T*4_*V*_]
Avg. duration (ms)	292, 125	305, 99	316, 104	333, 148	385, 172	397, 129	394, 141	429, 158	All
Avg. amplitude (°)	5.88, 3.08	6.48, 3.08	6.46, 2.42	6.78, 2.56	9.29, 3.56	9.78, 2.87	10.12, 3.58	10.47, 3.62	[*T*2_*F*_-*T*3_*F*_,*T*3_*F*_-*T*4_*F*_, *T*2_*V*_-*T*3_*V*_,*T*3_*V*_-*T*4_*V*_]
**Dynamic**									
Avg. K coeff.	0.75, 0.39	0.96, 0.61	0.92, 0.77	0.88, 0.89	0.16, 0.51	-0.06, 0.54	−0.19, 0.56	−0.31, 0.68	[*T*2_*F*_-*T*3_*F*_,*T*3_*F*_-*T*4_*F*_, *T*3_*V*_-*T*4_*V*_]
Avg. switch nb.	1.24, 0.26	1.46, 0.32	1.46, 0.31	1.47, 0.31	1.47, 0.29	1.65, 0.36	1.65, 0.34	1.63, 0.34	[*T*2_*F*_-*T*3_*F*_,*T*3_*F*_-*T*4_*F*_, *T*2_*V*_-*T*3_*V*_,*T*3_*V*_-*T*4_*V*_]

*The non-significant column regroups all post-hocs with a p-value > 0.05. Not mentioned post-hocs have a p-value <0.05 or less*.

#### 3.3.1. Number of Scrolls

During the trial, the participants scrolled on average 8.77 (*SD* = ±2.04) times. We found a task effect [*F*_(1,138)_ = 512.15, *p* < 0.001]. We measured lower numbers in the free viewing task (*M* = 6.62, *SD* = ±2.25) compared to the visual search task (*M* = 11.44, *SD* = ±2.63). We also found a time effect [*F*_(3,414)_ = 282.94, *p* < 0.001]. There was a significant increase between the first and second time-bins (*t* = 24.37, *p* < 0.001). However, there was no significant differences between the second and third time-bins (*t* = 0.19, *p* > 0.05) or between the third and fourth time-bins (*t* = −0.62, *p* > 0.05). There was a significant interaction between the time and task [*F*_(3,414)_ = 6.03, *p* < 0.001]. However, *post-hoc* analyses showed that the main effects were maintained (see Table 4).

#### 3.3.2. Scroll Duration

Scrolls lasted on average 367.57 (*SD* = ±121.65*ms*). We found a task effect [*F*_(1,138)_ = 205.20, *p* < 0.001]. Scroll was shorter in the free viewing task (*M* = 328.64*ms*, *SD* = ±99.57*ms*) compared to the visual search task (*M* = 417.24*ms*, *SD* = ±186.17*ms*). Additionally, we found a time effect [*F*_(3,414)_ = 55.49, *p* < 0.001]. There was a significant increase between the first and second time-bins (*t* = 9.34, *p* < 0.001), as well as between the third and fourth time-bins (*t* = 3.39, *p* < 0.01). However, there was no significant difference between the second and third time-bins (*t* = 1, *p* > 0.05). We did not find any interaction [*F*_(3,414)_ = 1.94, *p* > 0.05] (see Table 4).

#### 3.3.3. Scroll Amplitude

A scroll was on average 8.52°(*SD* = ±2.35) long. The task had an influence on scroll amplitude [*F*_(1,138)_ = 389.81, *p* < 0.001]. Scrolls were longer in the visual search task (*M* = 10.58°, *SD* = ±3.12°) than in the free viewing task (*M* = 6.91., *SD* = ±2.6.) The time also had an influence [*F*_(3,414)_ = 34.04, *p* < 0.001]. There was a significant increase between the first and second time-bins (*t* = 9.44, *p* < 0.001), but not between the second and third time-bins (*t* = 0.77, *p* > 0.05) or between the third and fourth time-bins (*t* = 1.20, *p* > 0.05). There was a significant interaction between the time and task [*F*_(3,414)_ = 6.51, *p* < 0.001], but *post-hoc* analyses confirmed that main effects were preserved (see Table 4).

#### 3.3.4. Scrolling Dynamic

In contrast to eye and mouse dynamics, scrolling dynamic was dominated by the focal mode (*M* = 0.43, *SD* = ±0.45). There was a task effect on the K coefficient [*F*_(1,138)_ = 454.64, *p* < 0.001], which was significantly more indicative of the focal mode in the free viewing task (*M* = 0.92, *SD* = ±0.67) than in the visual search task (*M* = −0.17, *SD* = ±0.47). There was also a time effect [*F*_(3,414)_ = 5.58, *p* < 0.001], the K coefficient significantly decreased between the first and second time-bins (*t* = −4.29, *p* < 0.001), but did not between the following successive time-bins (all *p > 0.05*). We found an interaction between the time and task [*F*_(3,414)_ = 39.55, *p* < 0.001]. The main task effect was maintained (all *p <0.001*). However, maintained during the free viewing task, the main time effect was not maintained in the visual search task. We measured a significant reduction between the first and second time-bins, and the second and third time-bins (all *p <0.05*, but not between the third and fourth time-bins (*p > 0.05*) (see Table 4).

#### 3.3.5. Modes Switches

The participants switched between modes an average of 3.63 (*SD* = ±0.74) times. There was a significant task effect [*F*_(1,138)_ = 257.59, *p* < 0.001]. The number of switches between modes was significantly lower in the free viewing task (*M* = 2.99, *SD* = ±0.94) than in the visual search task (*M* = 4.37, *SD* = ±1). We also found a significant time effect [*F*_(3,414)_ = 109.40, *p* < 0.001]. There was a significant decrease in the number of switches between the first and the second time-bins (*t* = −15.27, *p* < 0.001), but no significant differences between the following successive time-bins (all *p > 0.05*). The interaction of the time and task was also significant [*F*_(3,414)_ = 4.60, *p* < 0.001], but *post-hoc* analyses confirmed that main effects were preserved (see Table 4).

To summarize, we found a task and time effect for all scrolling parameters. As with the eyes and mouse parameters, most of the scrolling parameters stabilized at the end of the exploration. However, this evolution was in the opposite sense of that for the eye and mouse movements. While the eye and mouse fixation or dwelling parameters increased over time, scrolling dwells decreased. Inversely, while the eye and mouse movement parameters decreased over time, scrolling increased. As such, the focal mode was predominant in the global exploration, but tended to ambient mode over time.

## 4. Conclusion and Discussion

Since the seminal work of Buswell (1935), eye movements have been extensively studied in a wide variety of conditions. From viewing patterns (Yarbus, 1967) to average fixation durations (Mackworth and Morandi, 1967), how eye movement parameters behave are well-known. The knowledge of these basic parameters led to more complex research aiming to infer cognitive processes occurring during eye movements (Velichkovsky et al., 2002; Unema et al., 2005; Pannasch et al., 2008). However, with the stimuli diversity that aroused during the last decades, it became crucial to extend and adapt this knowledge to new stimuli types. That is why our study aims to provide a detailed statistical description of eye movement parameters on ecological web pages. Contrary to other stimuli such as natural images, web pages allow the use of mouse movements and scrolls. As previously described, mouse movements are mostly studied as patterns or trajectories (Rodden et al., 2008; Guo and Agichtein, 2010; Tzafilkou and Protogeros, 2018) and scrolling is sparsely studied (Braganza et al., 2009; Liu et al., 2017; Milisavljevic et al., 2018). Although their respective parameters are mentioned, to our knowledge, no quantitative analyses of their parameters have been performed. Using the same approach as for the study of eye movements, we intended to run such analyses to describe mouse and scroll parameters. Thus, the purpose of our study is to provide a statistics baseline of eye movements, mouse movements, and scrolling parameters during web pages exploration.

### 4.1. Eye Movement Parameters

We first found a task effect for all eye variables that replicated several studies in the literature (Yarbus, 1967; DeAngelus and Pelz, 2009; Itti and Borji, 2015). Fixation-related variables were higher in the free viewing task compared to the visual search task, while movement-related variables were higher in the visual search task. We also found a time effect on all variables. Fixation-related variables increased over time for both tasks while movement-related variables decreased. Participants did fewer fixations and saccades, but longer fixations and shorter saccades over time (Unema et al., 2005). As a result, we observed a global domination of ambient mode (i.e., short fixations with long saccades), but over time the dominant mode progressively switched to focal mode (i.e., long fixations with short saccades). This behavior could indicate that participants try to contextualize the stimulus at the beginning of the exploration to then focus more and more on content as time goes by.

### 4.2. Mouse Parameters

Then we ran the same analyses on mouse movements and scrolls. We found a task effect for all parameters of the mouse exploration, except for the average amplitude and duration of the mouse movements. As for the eye movements, dwell-related variables were higher in the free viewing task compared to the visual search task, while movement-related variables were higher in the visual search task. Again, we found a time effect on all variables. Comparably to eye movement parameters, dwell-related variables increased over time and movement-related variables decreased over time for both tasks. This behavior is similar to that of eye movements and suggests strong similarities between the two. Hence, we applied visual mode concepts to mouse movements. However, it is worth noting that the number of mouse movements was broadly lower to the number of eye movements, so these results should be discussed with caution. Despite the difference in the number of events, we observed similar behavior in the mouse dynamic, which began in ambient mode to progressively switch to focal model over the course of the exploration.

**Figure 3:**
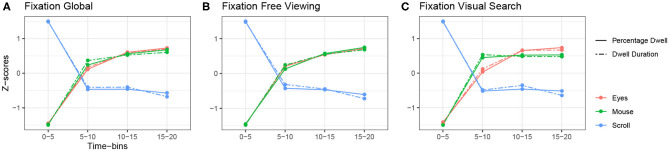
Relationship between fixation-related variables of the eyes, mouse and scroll. **(A)** Global z-scored averages. **(B)** z-scored averages over time in the free viewing condition. **(C)** z-scored averages over time in the visual search condition.

**Figure 4:**
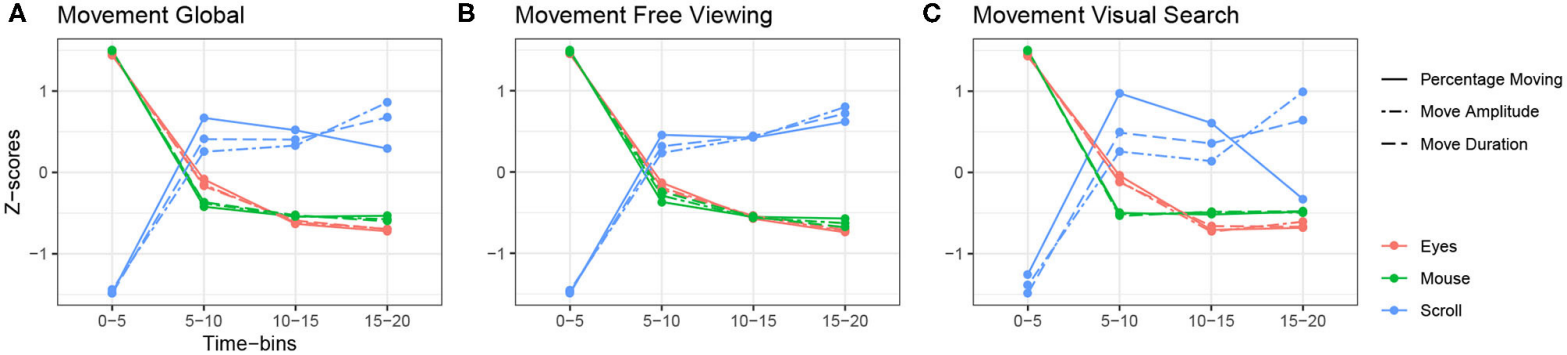
Relationship between saccade-related variables of the eyes, mouse and scroll. **(A)** Global z-scored averages. **(B)** z-scored averages over time in the free viewing condition. **(C)** z-scored averages over time in the visual search condition.

Regarding scrolling, all parameters varied according to the task. Comparably to eye and mouse movement parameters, we found a task effect for all parameters. We also found a time effect on all the variables, but dwell-related variables decreased over time while scroll-related variables increased. However, the stabilization of scroll parameters began earlier than for mouse parameters (see Figures 3, 4). Although there were fewer scroll movements than eye movements their frequency remained slightly higher than that of mouse movements. Therefore, we conducted analyses of dominant modes and found that, globally, scrolling was in focal mode. However, when looking over time, we observed that the focal mode was more dominant at the beginning of the exploration and ambient mode at the end. Since participants scrolled increasingly over time but did longer eye fixations, they seemed to balance the natural emergence of the focal mode of the eyes by scrolling to keep changing and contextualizing the newly displayed content.

### 4.3. Similarities and Differences

When studying eye, mouse, and scroll parameters, we observed common tendencies over time. In order to study these tendencies, we separated computed variables into two distinct categories: variables related to movements and variables related to fixations/dwells. Then, since focused on tendencies, the relevant parameters were normalized between 0 and 1 to enable the comparisons. This movement-fixation dichotomy is directly inspired from how the visual cortex processes visual information. The visual cortex is divided in two main pathways: ventral and dorsal stream. Ventral stream carries information about object recognition, while the dorsal stream is more related to visually guided movements (Goodale and Milner, 1992). Since saccades, mouse movements and scrolls are all visually guided movements, they are analyzed together. However, while fixation is directly involved in object recognition (ventral stream), it is not clear whether a mouse or a scroll dwell is involved. The mouse remains a tool used to browse a web page, and the implication of a pause still needs further investigations. For the convenience of the following analyses, we compare eye fixations with mouse and scroll dwells.

The first common tendency we observed is depicted in Figure 3A . It shows a common pattern between the fixation-related variables of the eyes and the mouse, and an opposite one with the scroll. On the one hand, eye and mouse parameters behaved similarly. Fixation or dwell durations, and percentages of fixations/dwells, were at their lowest at the beginning of the exploration and increased up to the end of exploration. For instance, in the free viewing task, the average fixation duration was 211.27 ms at the beginning of the exploration and increased up to 241.28 ms, while the average mouse dwell lasted 1 s at the beginning and increased up to 1.52 s (see Tables 2, 3 for more details). On the other hand, scrolling behaved exactly the opposite way. Scroll dwell was at its highest at the beginning of the exploration and lasted 1.91 s in average during the first time bin of the free viewing task and decreased overtime to reach 1.44 s at the end of the exploration (see Table 4 for more details). These observations are consistent in both the free viewing and visual search tasks (Figures 3B,C). Yet we observed a stabilization of mouse and scroll dwell durations starting from the second time-bin.

We observed a second tendency describing the opposite pattern for movement-related variables, as presented in Figure 4A. Eye and mouse movement variables decreased over time and scroll variables increased. Eye and mouse parameters behaved in the opposite way to scroll parameters, just as with fixation-related variables. Furthermore, this relationship was maintained across both tasks (Figures 4B,C). For instance, we observed an average saccade amplitude of 6.26°and an average mouse amplitude of 0.43°at the beginning of exploration during the free viewing task. Then both amplitudes have decreased to, respectively, 4.47°and 0.13°at the end of exploration. Under the same conditions, the scrolling amplitude increased from 5.88°at the beginning of the exploration to 6.78°at the end (see Tables 2–4 for more details).

Our results show a clear relationship between eye, mouse, and scroll parameters. Previous studies have already shown the spatial coordination of the eyes and mouse (Guo and Agichtein, 2010; Huang et al., 2012; Boi et al., 2016) and some coordination between the eyes and scroll speed (Milisavljevic et al., 2018). However, here we show that this relationship is even deeper than expected, and can be identified through analyzing eye, mouse, and scroll parameters. Indeed, coordination is not only between the eyes and the mouse, or, between the eyes and the scroll, but clearly between all three. Our findings show, for the first time, that eye and mouse parameters behave similarly, which confirms the interest of using mouse behavior to predict eye behavior. Yet the interaction described here does not take spatial coordinates into account that could be combined with relationship parameters to better predict eye movements from mouse events.

Even though further studies are needed to confirm our results, the relationship between eyes and mouse parameters seems consistent over time. This may be related to similar processing in the ventral and dorsal streams (Goodale and Milner, 1992). For instance, Stone and Gonzalez (2015) reported several studies in which ventral and dorsal streams of congenitally blind individuals were preserved during pointing and grasping tasks. Thus, we can assume that the important role of both streams involved in hand movements and eye movements may explain why the eyes and the mouse parameters behave similarly during the exploration. However, this hypothesis does not address why the scroll parameters behave oppositely. The opposite behavior we observed for the scroll may be explained by the “*the sensory weighting hypothesis*” (Ernst and Banks, 2002). This theory states that during a task involving sensory competition, here the presence of both vision and haptic, we tend to rely on the optimal one to complete the task. For instance, before reaching an object whose position is unknown, we first need to look at it, but there are occasions when we reach objects without looking at them because we already know their exact position. In our case, the task is to browse the page with or without a target. At the beginning of the exploration, the optimal sensory input to fulfill this task would be the eyes. As time goes by, we discover the web page more and more until we browsed it entirely. The scroll would gradually become the optimal way to browse the web page, since fixation duration is increasing and saccade amplitude decreasing, and the scroll would then replace large saccades.

Further research is necessary to better understand what mechanisms are involved in the eyes and mouse coordination during web pages exploration. For instance, we did not differentiate scroll up from scroll down in our analyses. When we scroll down, we usually discover the content for the first time. But a scroll up is necessary to re-examine an already seen area of the web page. Differentiating the two directions might provide finer results on what cognitive processes are involved.

## Data Availability Statement

The data analyzed in this study was obtained from the company Sublime Skinz. Data cannot be distributed, remixed, adapted, used to build upon, changed in any way or used commercially. Requests to access the datasets should be directed to Coralie Petermann, coralie@sublime.xyz.

## Ethics Statement

The studies involving human participants were reviewed and approved by Research Ethics Board of Paris Descartes University (Comité d'éthique de la Recherche de l'université Paris Descartes). The patients/participants provided their written informed consent to participate in this study.

## Author Contributions

AM, TL, MM, BG, and KD-M conceived and designed the study. AM, FA, and TL contributed to the data collection. AM and FA conducted all analyses. AM, FA, and KD-M wrote the manuscript. All authors contributed to the article and approved the submitted version.

## Conflict of Interest

The authors declare that the research was conducted in the absence of any commercial or financial relationships that could be construed as a potential conflict of interest.

## References

[B1] Anderson N. C. Ort E. Kruijne W. Meeter M. Donk M. (2015). It depends on when you look at it: Salience influences eye movements in natural scene viewing and search early in time. J. Vis. 15:9. 10.1167/15.5.9 26067527

[B2] Antal M. Egyed-Zsigmond E. (2019). Intrusion detection using mouse dynamics. IET Biometr. 8, 285–294. 10.1049/iet-bmt.2018.5126

[B3] Boi P. Fenu G. Spano L. D. Vargiu V. (2016). Reconstructing user's attention on the web through mouse movements and perception-based content identification. ACM Trans. Appl. Percept. 13, 1–21. 10.1145/2912124

[B4] Brady K. Cho S. J. Narasimham G. Fisher D. Goodwin A. (2018). Is scrolling disrupting while reading? in Proceedings of the 13th International Conference of the Learning Sciences (London), 8.

[B5] Braganza C. Marriott K. Moulder P. Wybrow M. Dwyer T. (2009). Scrolling behaviour with single- and multi-column layout, in Proceedings of the 18th international conference on World wide web - WWW '09 (Madrid: ACM Press), 831–840. 10.1145/1526709.1526821

[B6] Bruyer R. Abdi H. Benoit J. (1987). Stimulus versus face recognition in laterally displayed stimuli. Am. J. Psychol. 100, 117–121. 10.2307/1422645 3592025

[B7] Buscher G. Cutrell E. Morris M. R. (2009). What do you see when you're surfing? Using eye tracking to predict salient regions of web pages, in Proceedings of the SIGCHI Conference on Human Factors in Computing Systems - CHI '09 (Boston, MA: Association for Computing Machinery), 21–30. 10.1145/1518701.1518705

[B8] Buswell G. T. (1935). How People Look at Pictures: A Study of the Psychology and Perception in Art. Chicago, IL: University of Chicago Press.

[B9] Chen M. C. Anderson J. R. Sohn M. H. (2001). What can a mouse cursor tell us more? Correlation of eye/mouse movements on web browsing, in CHI '01 Extended Abstracts on Human Factors in Computing Systems, CHI EA '01 (New York, NY: Association for Computing Machinery), 281–282. 10.1145/634067.634234

[B10] Cronin D. A. Hall E. H. Goold J. E. Hayes T. R. Henderson J. M. (2020). Eye movements in real-world scene photographs: general characteristics and effects of viewing task. Front. Psychol. 10:2915. 10.3389/fpsyg.2019.02915 32010016 PMC6971407

[B11] DeAngelus M. Pelz J. B. (2009). Top-down control of eye movements: Yarbus revisited. Vis. Cogn. 17, 790–811. 10.1080/13506280902793843

[B12] Ernst M. O. Banks M. S. (2002). Humans integrate visual and haptic information in a statistically optimal fashion. Nature 415, 429–433. 10.1038/415429a 11807554

[B13] Feher C. Elovici Y. Moskovitch R. Rokach L. Schclar A. (2012). User identity verification via mouse dynamics. Inform. Sci. 201, 19–36. 10.1016/j.ins.2012.02.066

[B14] Ferreira S. Arroyo E. Tarrago R. Blat J. (2010). Applying Mouse Tracking to Investigate Patterns of Mouse Movements in Web Forms. Universitat Pompeu Fabra.

[B15] Fessenden T. (2017). Horizontal attention leans left.

[B16] Fu E. Y. Kwok T. C. Wu E. Y. Leong H. V. Ngai G. Chan S. C. (2017). Your mouse reveals your next activity: towards predicting user intention from mouse interaction, in 2017 IEEE 41st Annual Computer Software and Applications Conference (COMPSAC) (Turin: IEEE), 869–874. 10.1109/COMPSAC.2017.270

[B17] Gamboa H. Fred A. (2004). A behavioral biometric system based on human-computer interaction, in Biometric Technology for Human Identification, Vol. 5404 (Orlando, FL: International Society for Optics and Photonics), 381–392. 10.1117/12.542625

[B18] Goodale M. A. Milner A. D. (1992). Separate visual pathways for perception and action. Trends Neurosci. 15, 20–25. 10.1016/0166-2236(92)90344-8 1374953

[B19] Griffiths L. Chen Z. (2007). Investigating the differences in web browsing behaviour of chinese and european users using mouse tracking, in Usability and Internationalization. HCI and Culture, Vol. 4559 (Berlin; Heidelberg: Springer Berlin Heidelberg), 502–512. 10.1007/978-3-540-73287-7_59

[B20] Guo Q. Agichtein E. (2010). Towards predicting web searcher gaze position from mouse movements, in Proceedings of the 28th of the International Conference Extended Abstracts on Human Factors in Computing Systems - CHI EA '10 (Atlanta, GA: ACM Press), 3601. 10.1145/1753846.1754025

[B21] Helo A. Pannasch S. Sirri L. Rämä P. (2014). The maturation of eye movement behavior: scene viewing characteristics in children and adults. Vis. Res. 103, 83–91. 10.1016/j.visres.2014.08.006 25152319

[B22] Henderson J. M. (2003). Human gaze control during real-world scene perception. Trends Cogn. Sci. 7, 498–504. 10.1016/j.tics.2003.09.006 14585447

[B23] Henderson J. M. Hollingworth A. (1998). Eye movements during scene viewing: an overview, in Eye Guidance in Reading and Scene Perception, ed Underwood G. (Amsterdam: Elsevier), 269–293. 10.1016/B978-008043361-5/50013-4

[B24] Henderson J. M. Hollingworth A. (1999). High-level scene perception. Annu. Rev. Psychol. 50, 243–271. 10.1146/annurev.psych.50.1.243 10074679

[B25] Holmqvist K. Nyström M. Andersson R. Dewhurst R. Halszka J. van de Weijer J. (2011). Eye Tracking: A Comprehensive Guide to Methods and Measures. Oxford: Oxford University Press.

[B26] Huang J. White R. Buscher G. (2012). User see, user point: gaze and cursor alignment in web search, in Proceedings of the 2012 ACM annual conference on Human Factors in Computing Systems - CHI '12 (Austin, TX: ACM Press), 1341–1350. 10.1145/2207676.2208591

[B27] Itti L. Borji A. (2015). Computational models: bottom-up and top-down aspects. arXiv:1510.07748 [cs]. 10.1093/oxfordhb/9780199675111.013.026

[B28] Krejtz K. Duchowski A. Krejtz I. Szarkowska A. Kopacz A. (2016). Discerning ambient/focal attention with coefficient *K*. ACM Trans. Appl. Percept. 13, 1–20. 10.1145/2896452

[B29] Kumar M. Winograd T. Paepcke A. (2007). Gaze-enhanced scrolling techniques, in CHI'07 Extended Abstracts on Human Factors in Computing Systems (San Jose, CA), 2531–2536. 10.1145/1240866.1241036

[B30] Liu C. Liu J. Wei Y. (2017). Scroll up or down?: using wheel activity as an indicator of browsing strategy across different contextual factors, in Proceedings of the 2017 Conference on Human Information Interaction and Retrieval - CHIIR '17 (Oslo: ACM Press), 333–336. 10.1145/3020165.3022146

[B31] Mackworth N. H. Morandi A. J. (1967). The gaze selects informative details within pictures. Percept. Psychophys. 2, 547–552. 10.3758/BF03210264

[B32] Milisavljevic A. Bras T. L. Mancas M. Petermann C. Gosselin B. Doré-Mazars K. (2019). Towards a better description of visual exploration through temporal dynamic of ambient and focal modes, in Proceedings of the 11th ACM Symposium on Eye Tracking Research & Applications- ETRA '19 (Denver, CO: ACM Press), 1–4. 10.1145/3314111.3323075

[B33] Milisavljevic A. Hamard K. Petermann C. Gosselin B. Doré-Mazars K. Mancas M. (2018). Eye and mouse coordination during task: from behaviour to prediction, in Proceedings of the 13th International Joint Conference on Computer Vision, Imaging and Computer Graphics Theory and Applications (Funchal: SCITEPRESS - Science and Technology Publications), 86–93. 10.5220/0006618800860093

[B34] Moher J. Song J.-H. (2019). A comparison of simple movement behaviors across three different devices. Attent. Percept. Psychophys. 81, 2558–2569. 10.3758/s13414-019-01856-8 31493235

[B35] Mueller F. Lockerd A. (2001). Cheese: tracking mouse movement activity on websites, a tool for user modeling, in CHI'01 Extended Abstracts on Human Factors in Computing Systems (Seattle, WA), 279–280. 10.1145/634067.634233

[B36] Navalpakkam V. Jentzsch L. Sayres R. Ravi S. Ahmed A. Smola A. (2013). Measurement and modeling of eye-mouse behavior in the presence of nonlinear page layouts, in Proceedings of the 22nd International Conference on World Wide Web - WWW '13 (Rio de Janeiro: ACM Press), 953–964. 10.1145/2488388.2488471

[B37] Nielsen J. (2006). F-Shaped Pattern for Reading Web Content. Available online at: https://www.nngroup.com/articles/f-shaped-pattern-reading-web-content-discovered/

[B38] Nielsen J. (2010). Horizontal Attention Leans Left (Early Research). Available online at: https://www.nngroup.com/articles/horizontal-attention-original-research/

[B39] Pan B. Hembrooke H. A. Gay G. K. Granka L. A. Feusner M. K. Newman J. K. (2004). The determinants of web page viewing behavior: an eye-tracking study, in Proceedings of the 2004 Symposium on Eye Tracking Research & Applications (San Antonio, TX), 147–154. 10.1145/968363.968391

[B40] Pannasch S. Helmert J. R. Roth K. Herbold A.-K. Walter H. (2008). Visual fixation durations and saccade amplitudes: shifting relationship in a variety of conditions. J. Eye Mov. Res. 2. 10.16910/jemr.2.2.4

[B41] Pannasch S. Velichkovsky B. M. (2009). Distractor effect and saccade amplitudes: further evidence on different modes of processing in free exploration of visual images. Vis. Cogn. 17, 1109–1131. 10.1080/13506280902764422

[B42] Reeder R. Maxion R. (2006). User interface defect detection by hesitation analysis, in International Conference on Dependable Systems and Networks (DSN'06) (Philadelphia, PA: IEEE), 61–72. 10.1109/DSN.2006.71

[B43] Rheem H. Verma V. Becker D. V. (2018). Use of mouse-tracking method to measure cognitive load. Proc. Hum. Fact. Ergon. Soc. Annu. Meet. 62, 1982–1986. 10.1177/1541931218621449

[B44] Rodden K. Fu X. (2007). Exploring how mouse movements relate to eye movements on web search results pages, in 30th Annual International ACM SIGIR Conference (Amsterdam), 29–32.

[B45] Rodden K. Fu X. Aula A. Spiro I. (2008). Eye-mouse coordination patterns on web search results pages, in Proceeding of the Twenty-Sixth Annual CHI Conference Extended Abstracts on Human Factors in Computing Systems - CHI '08 (Florence: ACM Press), 2997–3002. 10.1145/1358628.1358797

[B46] Roth S. P. Tuch A. N. Mekler E. D. Bargas-Avila J. A. Opwis K. (2013). Location matters, especially for non-salient features-an eye-tracking study on the effects of web object placement on different types of websites. Int. J. Hum. Comput. Stud. 71, 228–235. 10.1016/j.ijhcs.2012.09.001

[B47] Seelye A. Hagler S. Mattek N. Howieson D. B. Wild K. Dodge H. H.. (2015). Computer mouse movement patterns: a potential marker of mild cognitive impairment. Alzheimer's Dement. 1, 472–480. 10.1016/j.dadm.2015.09.006 26878035 PMC4748737

[B48] Sharmin S. Špakov O. Räihä K.-J. (2013). Reading on-screen text with gaze-based auto-scrolling, in Proceedings of the 2013 Conference on Eye Tracking South Africa, ETSA '13 (New York, NY: Association for Computing Machinery), 24–31. 10.1145/2509315.2509319

[B49] Still J. D. Masciocchi C. M. (2010). A saliency model predicts fixations in web interfaces, in Proceedings of the 5th International Workshop on Model-Driven Development of Advanced User Interactions, Vol. 617 (Atlanta, GA), 25–28.

[B50] Stone K. D. Gonzalez C. L. R. (2015). The contributions of vision and haptics to reaching and grasping. Front. Psychol. 6:1403. 10.3389/fpsyg.2015.01403 26441777 PMC4584943

[B51] Tatler B. W. Vincent B. T. (2008). Systematic tendencies in scene viewing. J. Eye Mov. Res. 2, 1–18. 10.16910/jemr.2.2.5

[B52] Theeuwes J. Failing M. (2020). Attentional Selection: Top-Down, Bottom-Up and History-Based Biases. Elements in Perception. Cambridge: Cambridge University Press. 10.1017/9781108891288

[B53] Torralba A. Oliva A. Castelhano M. S. Henderson J. M. (2006). Contextual guidance of eye movements and attention in real-world scenes: the role of global features in object search. Psychol. Rev. 113:766. 10.1037/0033-295X.113.4.766 17014302

[B54] Tullis T. S. (2007). Older adults and the web: lessons learned from eye-tracking, in Universal Acess in Human Computer Interaction. Coping with Diversity, Vol. 4554 (Berlin; Heidelberg: Springer Berlin Heidelberg), 1030–1039. 10.1007/978-3-540-73279-2_115

[B55] Tzafilkou K. Protogeros N. (2018). Mouse behavioral patterns and keystroke dynamics in end-user development: what can they tell us about users' behavioral attributes? Comput. Hum. Behav. 83, 288–305. 10.1016/j.chb.2018.02.012

[B56] Unema P. J. A. Pannasch S. Joos M. Velichkovsky B. M. (2005). Time course of information processing during scene perception: the relationship between saccade amplitude and fixation duration. Vis. Cogn. 12, 473–494. 10.1080/13506280444000409

[B57] Velichkovsky B. M. Rothert A. Kopf M. Dornhöfer S. M. Joos M. (2002). Towards an express-diagnostics for level of processing and hazard perception. Transport. Res. Part F Traff. Psychol. Behav. 5, 145–156. 10.1016/S1369-8478(02)00013-X

[B58] Yarbus A. L. (1967). Eye Movements and Vision. New York, NY: Plenum. 10.1007/978-1-4899-5379-7

[B59] Zheng N. Paloski A. Wang H. (2011). An efficient user verification system via mouse movements, in Proceedings of the 18th ACM Conference on Computer and Communications Security - CCS '11 (Chicago, IL: ACM Press), 139–150. 10.1145/2046707.2046725

